# Inhibition of EGR1 inhibits glioma proliferation by targeting CCND1 promoter

**DOI:** 10.1186/s13046-017-0656-4

**Published:** 2017-12-15

**Authors:** Dian-gang Chen, Bo Zhu, Sheng-qing Lv, Hongfan Zhu, Jinliang Tang, Changlin Huang, Qingrui Li, Pu Zhou, Dong-lin Wang, Guang-hui Li

**Affiliations:** 1 Institute for Cancer Research in People’s Liberation Army, Xinqiao Hospital, Third Military Medical University, Chongqing, 400037 China; 2 Department of Pathology, Xinqiao Hospital, Third Military Medical University, Chongqing, 400037 China; 3 Department of Neurosurgery, Xinqiao Hospital, Third Military Medical University, Chongqing, 400037 China; 4 Department of Oncology, Cancer Hospital of Chongqing City, Chongqing, 400037 China

**Keywords:** EGR1, CCND1, Glioma, Proliferation

## Abstract

**Background:**

Gliomas are the most common primary tumors in central nervous system. The prognosis of the patients with glioma is poor regardless of the development of therapeutic strategies. Its aggressive behavior mainly depends on the potent ability of proliferation. The transcription factor EGR1 (early growth response 1) is a member of a zinc finger transcription factor family which plays an essential role in cell growth and proliferation.

**Methods:**

EGR1 expression levels in 39 glioma tissues and 10 normal brain tissues were tested by RT-qPCR and Western-blotting. The effects of EGR1 on U251 cells, U251 stem-like cells (GSCs), and U87 cells proliferation were assessed using in vitro and in vivo cell proliferation assays. The specific binding between EGR1 and CCND1 promoter was confirmed by CHIP assay. EGF was used to improve EGR1 expression in this assay.

**Results:**

EGR1 expression levels in human gliomas are decreased compared with normal brain tissues, however, the patients with low EGR1 expression level showed significantly enhanced patient survival in all glioma patients. EGR1 silencing inhibited proliferation and induced G1 phase arrest in glioma cells. EGR1 contributed to proliferation by directly raising CCND1. Meanwhile, EGR1 overexpression induced by EGF was able to promote the proliferation of glioma cells.

**Conclusions:**

Our results show that stable knockdown EGR1 would inhibit glioma proliferation. The results suggest EGR1 showing lower expression in cancer tissues compared with normal tissues maybe still play an important role in tumor proliferation.

**Electronic supplementary material:**

The online version of this article (10.1186/s13046-017-0656-4) contains supplementary material, which is available to authorized users.

## Background

According to the American Brain Tumor Association, gliomas represent 24.7% of all primary brain tumors and 74.6% of all malignant tumors. According to the current WHO classification, astrocytomas are divided into four histological grades [1]. Grades include low-grade, or WHO grade I (pilocytic astrocytoma) and grade II (diffuse astrocytoma); and high-grade, or WHO grade III (anaplastic astrocytoma) and grade IV (glioblastoma multiforme, GBM). Grade III and IV tumors are considered malignant gliomas. Glioblastoma represent 14.9% of all primary brain tumors, and 55.4% of all gliomas. Glioblastoma has the highest number of cases of all malignant tumors, with an estimated 12,390 new cases predicted in 2017. Glioblastoma have the most aggressive clinical course (median survival between 14.5 and 16.6 months) [2] [3]. Despite current therapy consisting of surgery followed by radiation and temozolomide has a moderate success rate and the tumor reappears with an average patient survival of around 15 months [4]. Using of low-intensity, intermediate-frequency alternating electric fields (tumor treating fields, or TTF), in conjunction with standard chemoradiotherapy to treat patients is able to increase the overall survival time to 19.4 months in a phase III trial in patients with newly diagnosed with glioblastoma [5]. However, surviving patients with glioma often suffer devastating long-term side effects induced by a series of therapies. The patients with GBM still suffered the worst prognosis and serious adverse effect caused by lesion and therapy. So, a better understanding of the molecular mechanisms of the disease remains essential for the development of new therapeutic strategies.

EGR1 (early growth response 1), also known as NGFI-A, KROX-24, ZIF268, and TIS8, is a member of the early growth response (EGR) family. The expression of EGR1 is stimulated by many extracellular signaling molecules, including hormones, neurotransmitters, growth and differentiation factors, and cytotoxic metabolites [6]. Its biological role has been linked to several key cellular functions, such as proliferation [7], apoptosis [8], and migration [9]. Intriguingly, EGR1 can function as a tumor suppressor or an oncogene, depending on the type of tumor cells. In the prostate tumor, EGR1 stimulates cell growth [10]. Contrarily, EGR1 expression is often absent or reduced in breast cancer, which also results in tumor growth [11]. The expression of EGR1 is also decreased in human GBM compare to normal brain tissue [12], but the effect of EGR1 on glioma cell proliferation is still paradoxical. Michel Mittelbronn et al. showed that EGR1 expression was significantly associated with enhanced patient survival and was an independent prognostic factor in multivariate analysis in high grade astrocytomas [13]. In contrast, Nathalie Sakakini et al. found that a positive feed-forward loop associating EGR1 and PDGFA promotes proliferation and self-renewal in Glioblastoma Stem Cells (GSCs) [14]. Because of its paradoxical function in gliomas, further elucidation of its mechanism of EGR1 regulating the proliferation remains essential.

We showed here that the expression of EGR1 is reduced in human glioma tissues compare to normal brain tissues, which is consistent with the result of TCGA. But, stable knockdown of EGR1 in GSCs and normal glioma cells inhibited growth in cellular level and xenografted tumor. EGR1 contributed to proliferation by directly transcript CCND1 gene that involved in G0/G1 phase regulation. Moreover, the growth factor EGF stimulated glioma cells proliferation partially by enhancing EGR1 expression.

## Methods

### Cell culture

Glioma samples were obtained from consenting patients, as approved by the Research Ethics Boards at The Xinqiao Hospital. Glioma samples included low-grade astrocytomas (19 cases) and high-grade astrocytomas (20 cases). All of the samples were frozen in liquid nitrogen. Human GBM cell lines (U87 and U251) were purchased from Cell Bank of Chinese Academy of Sciences. The cells were cultured in Dulbecco’s Modified Eagles Medium high glucose (DMEM) (Gibco, Carlsbad, CA, USA) supplemented with 10% fetal bovine serum (FBS) (Gibco, Carlsbad, CA, USA) and 1% Penicillin streptomycin (Gibco, Carlsbad, CA, USA) at 37 °C in a humidified atmosphere containing 5% CO_2_.

U251 stem-like cells (U251SLC) were induced according to the manipulation established by our lab [15]. Briefly, U251 glioblastoma cells were seeded in 6-well plates containing 2 ml DMEM, supplemented with 10% fetal bovine serum (FBS) (Gibco, USA) overnight. Thereafter, culture medium was replaced with 2 ml serum-free neural stem cell medium containing DMEM/F12 (Gibco), B27 (1X, Gibco), 20 ng/ml basic fibroblast growth factor (bFGF; peprotech), 20 ng/ml epidermal growth factor (EGF; peprotech), insulin (4 U/l; Sigma). This procedure was repeated every 24 h until several primary tumor spheres were visible under microscopy (about 4–5 weeks). At this point, all culture medium was discarded and the cells were moved into glass flasks with 10 ml fresh serum-free neural stem cell medium. The expression of CD133 was analyzed by flow cytometry.

### Virus production and transduction

The EGR1 knockdown lentiviral vector (siEGR1, CAACGAGAAGGTGCTGGTG) was constructed by Shanghai GeneChem Co., Ltd. (Shanghai, China). A GFP lentiviral vector was used as negative control (NC). All lentiviral vectors expressed GFP and puromycin, which enabled us to select stably transfected cells. The day before transfection, cells were seeded in 24-well plates at a density of 50,000 cells per well. The lentivirus transfection was performed according to the manufacturer’s instruction, with MOI(multiplicity of infection) = 10, and stably transfected cells were selected by puromycin (5 μg/ml).

### RNA isolation and quantitative real-time PCR

Total RNA was isolated from cells using RNAiso Plus (TaKaRa). For complementary DNA (cDNA) synthesis, 1 μg of total RNA was reverse transcribed using the PrimeScript™ RT Reagent Kit (TaKaRa) and gDNA Eraser (Perfect Real Time) (TaKaRa) and carried out in triplicate with an ABI 7500 Prism Sequence Detection System (Applied Biosystems, Foster City, CA). The amplification conditions were as follows: 95 °C for 30 s, followed by 40 cycles of: 95 °C for 5 s, 60 °C for 34 s. For normalization of all RT-qPCR data, β-ACTIN expression was used as a reference gene. Primers used in real-time qPCR were as follows: β-ACTIN, forward: 5’-GTGAAGGTGACAGCAGTCGGTT-3′, reverse: 5’-GAAGTGGGGTGGCTTTTAGGA-3′; EGR1, forward: 5’-CAGCACCTTCAACCCTCAG-3′, reverse: 5’-CACAAGGTGTTGCCACTGTT-3′; CCND1, forward: 5’-TATTGCGCTGCTACCGTTGA-3′, reverse: 5’-CCAATAGCAGCAAACAATGTGAAA-3′.

### Western blotting analysis

To examine the protein level of EGR1, CCND1, cells were collected and lysed on ice for 10 min in RIPA Lysis Buffer (Beyotime, Jiangsu, China) with protease inhibitor phenylmethanesulfonyl fluoride (PMSF, Beyotime, Jiangsu, China). 20 μg of total protein from each sample was separated on 10% polyacrylamide gels (Beyotime, Jiangsu, China). After electrophoresis, separated proteins were transferred onto polyvinylidene fluoride (PVDF) membranes (Roche Applied Science). Membranes were subsequently blocked for 1 h at room temperature with 5% BSA (BOSTER, AR0004). The PVDF membranes were, respectively, incubated over night with the mouse monoclonal anti-β-ACTIN (dilution 1:1000; BOSTER, BM0627), rabbit polyclonal anti-EGR1 (dilution 1:1000; santa cruz, sc-110×), mouse monoclonal anti-CCND1 (1:3000; Abcam, ab134175). After washing with TBST, membranes were probed with goat anti-rabbit IgG (dilution 1:5000) or goat anti-mouse IgG (dilution 1:5000) conjugated with HRP for 1 h at room temperature. Labeled bands were detected by BeyoECL Plus (Beyotime, Jiangsu, China). Results expressed relative to β-ACTIN band density used as a loading control.

### Chromatin immunoprecipitation (ChIP)

10^7^ cells were used per ChIP assay. ChIP was performed using the EZ-ChIP Kit (Millipore) according to the manufacturer’s instructions. DNA was sheared with six 10-s “on” and 30-s “off” pulses in iced water using a sonicator 2-mm tip set to 30% amplitude. Chromatin was sonicated to an average fragment size of 200 bp–800 bp. A fraction (1%) of the sonicated chromatin was used as ‘input’ DNA and the RT-qPCR results were analyzed using the Percent Input Method (Invitrogen, Carlsbad, CA, USA). Briefly, the percent input was calculated by the formula: 100 × 2^(adjusted input Ct-IP Ct). The threshold cycle (Ct) value of input, which is 1% of the immunoprecipitation (IP) reaction, was adjusted to 100% by subtracting 6.644 cycles (log2 of 100). Antibodies used for immunoprecipitation were EGR1 (santa cruz, sc-110×). Primers used were: GAPDH promoter, forward: 5’-TACTAGCGGTTTTACGGGCG-3’and reverse: 5’-TCGAACAGGAGGAGCAGAGAGCGA-3′; CCND1 promoter, forward: 5’-CTCTGCCGGGCTTTGATCTT-3′ and reverse: 5’-ATGGTTTCCACTTCGCAGCA-3′.

### Proliferation and survival assays.

The cell proliferation was assessed by Cell Counting Kit-8 (CCK-8, Dojindo, Kumamoto, Japan). Cells were seeded in 96-well plates at 3000 cells per well. After 10 μl of CCK-8 reagents were added, the cells were continuously incubated for 2 h. The spectrophotometric absorbance of the samples was measured with a microplate reader iMARK (Bio-Rad, Hercules, CA, USA) at 450 nm with a reference wavelength of 630 nm. All experiments were repeated three times.

iClick™ EdU Andy Fluor™ 647 Flow Cytometry Assay Kit (GeneCopoeia, USA) was used to evaluate the proliferation of glioma cells. All cells were treated with 40 μM EdU and detected according to the recommended staining protocol. U251 cells and U87 cells cultured in 10% FBS medium treated with EdU for 6 h. For observing the effect of EGF on proliferation of U251 cells and U251SLCs with/without EGR1 RNAi, the cells were seeded in 6 well culture-plates in the DMEM supplemented with 10% FBS over one night, replace medium with 0.5% FBS for 24 h. Then, EGF was added to the culture medium concomitance with EdU and the final concentration of EGF was 20 ng/ml. The cells were collected and flow cytometry assay was performed to detect the proliferation of U251 cells and U251SLCs with/without EGR1 RNAi after dealt with EGF and EdU for 12 h. Flow cytometry assay followed the instructions.

### Cell cycle analysis

Cells were collected and fixed with ice-cold 70% ethanol overnight at 4 °C. The fixed cells were stained with 0.5 mL of propidium iodide (PI) staining buffer (contains 200 mg/mL RNase A and 50 μg/mL PI) at room temperature for 30 min in dark. PI-stained cells were analyzed by flow cytometry (BD Biosciences, Franklin Lakes, NJ, USA).

### Mouse injections and tumor assays

U251 cells or U251 stem-like cells were dissociated into single-cell suspensions in serum-free, antibiotic-free medium. One million (U251SCLs) or five million (U251) cells were injected subcutaneously into 20 six-week-old male SCID mice divided into 4 groups. Mice implanted with U251 cells or U251SLC were sacrificed at the 50th day and the 40th day after implantation, respectively. The tumor tissues were fixed for pathological review. The tissue sections were stained by hematoxylin and eosin and human-specific antibodies against GFAP (Zhongshan Biotechnology, China). The volume of the tumor was calculated according to the formula: V = (length × width^2^)/2 [16]. All the animal experiments were in strict accordance with the Institutional Animal Care guidelines of Third Military Medical University.

### Immunohistochemistry

Immunohistochemical (IHC) analysis was conducted to study GFAP, Ki-67, EGR1, and CCND1 protein expression in glioma xenografts. Briefly, fresh glioma xenografts were fixed in 4% paraformaldehyde, embedded in paraffin, and cut into 5-um-sections. Then, the sections were immunohistochemically stained using Ki-67 antibody (1:100, proteintech, 27,309–1-AP). EGR1 antibody (dilution 1:100; santa cruz, sc-110×), CCND1 antibody (1:100; Abcam, ab134175). Slides were imaged under a light microscope (Leica).

### TCGA data analyses

To analyze differential EGR1 expression between normal brain tissues and glioma tissues, we generated EGR1 differential plot in web of http://firebrowse.org/. First, input “EGR1” in View Expression Profile box, then, choose “Filter on” and “GBMLGG” (lower grade glioma and glioblastoma), submit.

To analyze the effect of EGR1 expression on prognostic of glioma patients, we generated Kaplan-Meier survival curve of GBMLGG patients with low or high expression of EGR1 by using Kaplan-Meier Plotter (https://xenabrowser.net/heatmap/#). Specifically, select “Visualization” at the top of the web of https://xenabrowser.net/heatmap/#, choose “TCGA lower grade glioma and glioblastoma (GBMLGG)” in “Cohort” drop-down list. Then, choose “+Date”, “gene expression RNAseq”, “gene expression RNAseq (polyA+ IlluminaHiSeq)”, “next” in turn. Then, input “EGR1” into Genes box, “Done”. Then, choose “Column menu (Inverted triangle symbol)”, “Kaplan Meier Plot”.

### Statistical analyses

Statistical analyses for TCGA are described above. Statistical analyses for functional and biochemical in vitro and in vivo studies were performed using two-tailed distribution unpaired Student t-test. All dot plots were generated by Graphpad Prism 5. All histograms were presented as mean ± SEM. *P* values of equal or less than 0.05 were considered significant and were marked with an asterisk(*) on the histogram. P values of equal or less than 0.01 were denoted by **, and P values of equal or less than 0.001 were denoted by *** on the histograms.

## Results

### Expression of EGR1 in GBMLGG

**Fig. 1:**
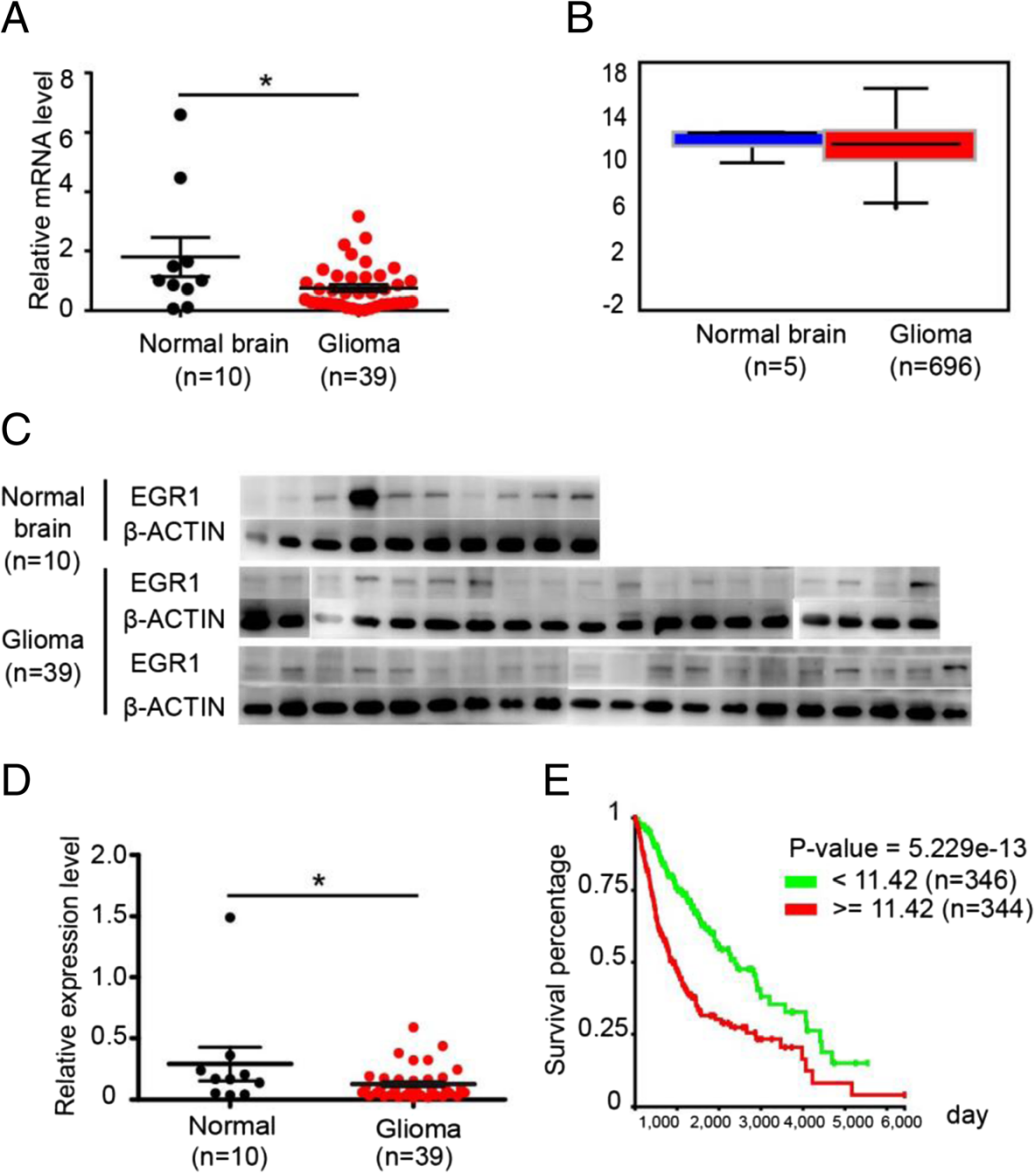
Expression of EGR1 in GBMLGG. **a** The mRNA levels of EGR1 in giloma tissues and normal brain tissues. *P* = 0.024. **b** The mRNA levels of EGR1 in giloma tissues and normal brain tissues, data come from TCGA databases. **c** Immunoblot analysis of EGR1 total protein levels in glioma tissues and normal brain tissues. **d** Relative protein levels of EGR1 were determined by Western blotting. The levels of EGR1 were normalized to those of β-ACTIN. *P* = 0.0447. **e**. Kaplan-Meier analysis of the GBMLGG RNA-seq data were from the The Cancer Genome Atlas (TCGA) databases. *P < 0.05, (mean ± SEM, Student’s t-test)

Aggressive tumors often possess the characters of infiltration and fast growth. To assess whether EGR1 might be associated with the malignancy of glial tumors, the expression of EGR1 was compared between normal brain tissues (NBTs) and glial tumors (GTs)**.** We performed real-time qPCR of EGR1 mRNA expression in 10 NBTs and 39 GTs. The results revealed that the EGR1 mRNA expression levels in GTs were lower as compared with that in NBTs (*p* = 0.024) (Fig. 1a), but no significant difference of EGR1 mRNA expression levels was observed between NBTs and GTs groups in the The Cancer Genome Atlas (TCGA) database (Fig. 1b) and Western-blotting results, lower EGR1 protein in GTs compared with NBTs (*p* = 0.0447) (Fig. 1c-d). All the results both in mRNA levels and protein levels are similar to the report showed by Antonella Calogero et al. [12], who reported that EGR1 mRNA was markedly down-regulated in astrocytomas and in glioblastomas versus normal brain. Furthermore, Michel Mittelbronn et al. showed EGR1 expression was significantly decreased and associated with enhanced patient survival and was an independent prognostic factor in multivariate analysis in high grade astrocytomas [13]. But, the result of their studies conflicts with the result from the TCGA database. Kaplan-Meier analysis using the The Cancer Genome Atlas (TCGA) database showed that lower EGR1 expression provided a better patient outcome between the different EGR1 gene expression subtypes (*P* < 0.001) (Fig. 1f). Of interest, we found the expression level of EGR1 in glioma stem-like cells was sustaining higher than that in normal glioma cells (Additional file 1: Figure S1A). Compared with normal glioma cells, Glioma stem-like cells always show stronger invasion and proliferation ability. So, we wondered if stably alter EGR1 expression levels would influence glioma proliferation. Then, expression of EGR1 gene was knocked down by RNAi in several glioma cell lines.

### EGR1 silencing inhibits proliferation and induces G1 phase arrest in glioma cells

**Fig. 2:**
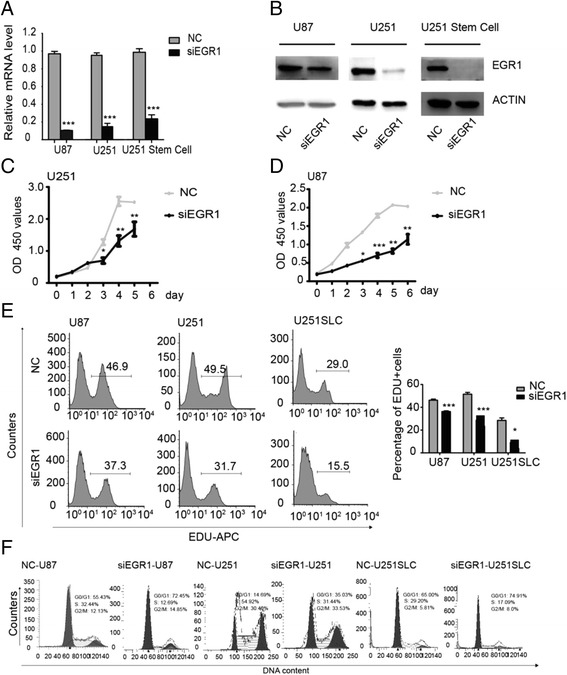
Targeting EGR1 by RNA interference inhibited the proliferation of glioma cells. **a** Real-time quantitative PCR analysis of EGR1 in U251, U251SLC, and U87 cells transfected with control siRNA vector and siEGR1-vector. **b** Immunoblot analysis of EGR1 in U251, U251SLC, and U87 cells transduced with control vector and siEGR1-vector. **c**, **d** The rates of cell growth were detected by CCK8 assay in U87 and U251 cells transfected with control siRNA or EGR1 siRNA. **e** Cell proliferation rates as determined by EDU assay in U87, U251 and U251SLC cells transfected with control siRNA or EGR1 siRNA. **f** Cell cycle analyzed by propidium iodide staining and flow cytometry in U87, U251 or U251SLC cells transfected with control siRNA or siRNA targeting EGR1. (At least three repeated experiments for the all cell types). *P < 0.05, **P < 0.01, and ***P < 0.001 (mean ± SEM)

To determine whether EGR1 expression decreasing would induced the proliferation suppressing of glioma cell, the EGR1 RNA interference (RNAi) in glioma cell lines (U87 and U251) and one stem-like cell line (U251stem-like cell) were performed. U251SLC was induced from the U251 cell lines according to the manipulation established by our laboratory [15]. The U251SLC was identified using CD133 marker and clonogenic ability (Additional file 1: Figure S1B-E). The expression of EGR1 was knocked down by a lentiviral siRNA (siEGR1). EGR1 mRNA and protein levels of the three cell lines were significantly reduced compared with the control group (Fig. 2a-b). These results indicated that the specific siRNA targeting EGR1 was able to effectively knockdown endogenous EGR1 at both mRNA and protein levels in U87, U251 and U251 stem-like cells.

CCK-8 and EDU (5-Ethynyl-2′-deoxyuridine) assays were performed to assess cell proliferation. Results of CCK8 assay showed that the cell proliferation was inhibited in siEGR1-U251 cells and siEGR1-U87 cells compared with control group (Fig. 2c-d). Because of clustering growth of U251SLC, the number of cells can’t be accurately reflected by CCK-8 assay. The proliferation for U251SLC was detected by EDU (5-Ethynyl-2′-deoxyuridine) assay instead of CCK8 assay. In addition, EDU-positive cell rates were significantly decreased in siEGR1 group compared to the negative control (NC) group (Fig. 2e). These results identified the EGR1 knockdown by RNAi inhibited the proliferation of U251, U87 and U251SLC cells.

To understand the mechanisms of cell proliferation suppressed, the percentages of cells in different phases of the cell cycle were analyzed by flow cytometry. A significant decreases in S phase was observed in siEGR1 group (12.69% in U87, 31.44% in U251, 17.09% in U251SLC), compared with the NC group (34.60% in U87, 54.92% in U251, 29.20% in U251SLC) (Fig. 2f). At the same time, A significant increase in S phase was observed in siEGR1 group (72.45% in U87, 35.03% in U251, 74.91% in U251SLC), compared with the NC group (55.43% in U87, 14.69% in U251, 65.00% in U251SLC) (Fig. 2f). These data demonstrated that knockdown of EGR1 lead to G1 phase arrest and inhibited glioma cell proliferation.

### EGR1 silencing inhibits the proliferation of U251 cell and U251SLCs through direct downregulating CCND1

**Fig. 3:**
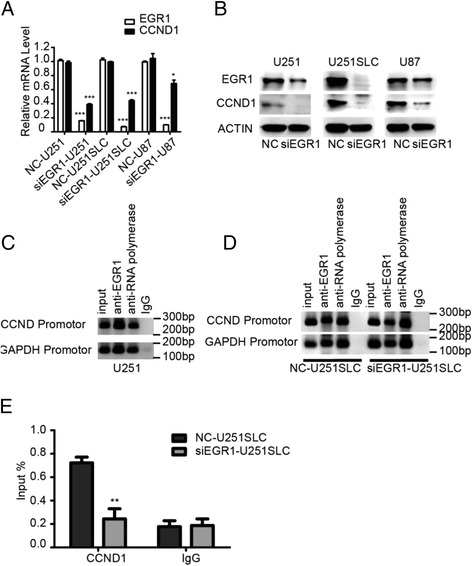
CCND1 expression was regulated by EGR1 that directly bound in the promotor of CCND1 in glioma cells. Lysates of U87, U251 and U251SLC cells expressing NT control siRNA, siEGR1 were analyzed by real-time quantitative PCR (**a**) and by immunoblotting(**b**). siRNA-mediated knockdown of EGR1 inhibits CCND1 expression. **c** Chromatin fragments from U251 cells were immunoprecipitated (with antibodies specific to RNA polymerase II (anti- RNA polymerase II; positive control), mouse IgG (IgG, negative control), and EGR1 (anti-EGR1) as indicated. Input, 1% total DNA. After reversal of cross-linking, the immunoprecipitated DNA was amplified by PCR using the specific primers and resolved on 2% agarose gels. ChIP assay demonstrated that EGR1 protein bound to the promoter region of the CCND1 gene. **d** The immunoprecipitated DNA of U251SLC cells expressing negative control siRNA and siEGR1 was amplified by PCR using the specific primers and resolved on 2% agarose gels. **e** RT-qPCR analyses the immunoprecipitated DNA of NC-U251SLC and siEGR1-U251SLC. Values were expressed relative to percent input. *P < 0.05, **P < 0.01, and, ***P < 0.001 (mean ± SEM)

To address the mechanisms responsible for EGR1-silencing–mediated inhibition of cell proliferation, we examined the status of intracellular signaling molecules. EGR1 activates a number of genes containing the NAB1, NAB2, P53, IL-2, Igf2, PDGF-A, TGF-β, CCND1 and so on [17–19]. Since CCND1 is one of the molecules which regulate the process from the G1 phase into the S phase, we hypothesized that CCND1 may be regulated by EGR1 in glioma. The results showed that silencing of EGR1 reduced CCND1 in both mRNA (Fig. 3a) and protein levels (Fig. 3b).

In order to provide a direct link between EGR1 and CCND1, we investigated whether EGR1 was able to bind to the promotor region of the CCND1 gene. The CCND1 promotor sequence was obtained from the web (http://epd.vital-it.ch/human/human_database.php). The whole 700 bp CCND1 promotor sequence (−500 to 200) (Additional file 1: Figure S2A) was analyzed in the web (http://jaspar.genereg.net/) using “JASPAR CORE Vertebrata” and the “relative profile score threshold” was 95%. A potential EGR1 binding site (−121 to-108) in this sequence was found (Additional file 1: Figure S2A). According to the sequence, we designed a pair of primers (Additional file 1: Figure S2A). Following the EZ-CHIP instructions, chromatin was sonicated to an average fragment size of 200 bp–800 bp (Additional file 1: Figure S2B). 60s was chose in this assay. The cross-linked and sonicated human chromatins prepared from U251 cells or U251SLCs were immunoprecipitated with antibodies specific for either EGR1 or RNA polymerase II. Normal mouse IgG was used as a negative control. The genomic DNA associated with the immunoprecipitated chromatin was amplified by RT-qPCR. The results identified that anti-EGR1 antibody precipitated the CCND1 promotor fragment in U251 cell lines (Fig. 3c), which confirmed that the CCND1 sequence contains EGR1 binding sequence (CGCCCGCCCCCGCC) (Additional file 1: Figure S2A). The EGR1 binding site in CCND1 gene promotor region is located at −122 bp to −109 bp and the TATA box of CCND1 gene banded by RNA polymerase II antibody which located at −241 bp to −225 bp. There was only ~100 bp between the EGR1 binding site and TATA box in CCND1 promotor (Additional file 1: Figure S2A). Similar status also presents in the GAPDH promotor region. The GAPDH promotor region was analyzed and we also found an EGR1 binding site (−432 to −419) near to site of TATA-box (−681 to −656) in GAPDH gene which was able to bind by RNA polymerase II antibody (data not given). Because both the CCND1 promotor and GAPDH promotor contain EGR1 binding sequence and TATA-box and the two site are close to each other, both target sequence containing EGR1 binding sequence and TATA-box in CCND1 gene or GAPDH gene, banded by anti-RNA polymerase II antibody or anti-EGR1 antibody, were comprised of part of CCND1 and GAPDH promotor. It resulted in the band of CCND1 and GAPDH promotor all appearing on target sequence banded by both anti-RNA polymerase II antibody and anti-EGR1 antibody (Fig. 3c).

In order to further confirming the binding of EGR1 to CCND1 promotor, the immunoprecipitated DNA of U251SLC cells expressing negative control siRNA (NC-U251 SLC) and U251SLC cells expressing siEGR1 (siEGR1-U251SLC) was amplified by PCR using the specific primers and resolved on 2% agarose gels. The results of PCR showed fewer binding in siEGR1-U251SLC compared it to NC-U251SLC (Fig. 3d). Real-time PCR showed similar results in siEGR1-U251SLC cells and NC-U251SLC cells (Fig. 3e). These data indicated that EGR1 transcriptionally regulated CCND1 expression to promote the growth of glioma cells.

### EGR1 is required for glioma cells proliferation in mouse xenograft model of U251 cells and U251SLCs

**Fig. 4:**
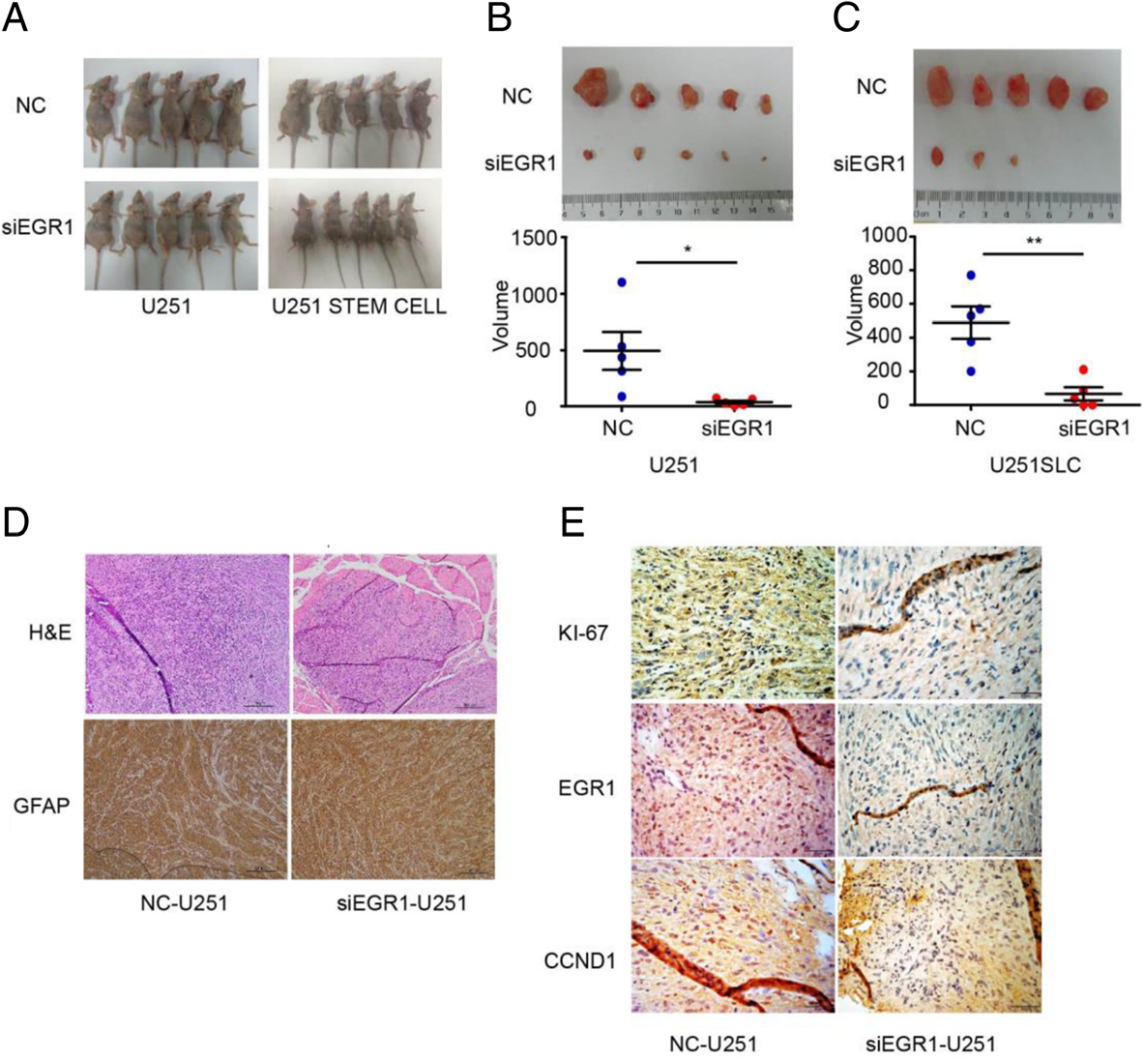
EGR1 RNAi suppressed the growth of glioma xenograft tumor in vivo. **a** siEGR1-U251 cells (5 × 10^6^) and NC-U251 (5 × 10^6^) cells were inoculated subcutaneously into armpit of BALB/C nude mice, all of the mice examined developed tumors at 50th day. In NC-U251SC (1 × 10^6^) group, all mice developed xenograft tumors at 40th day. In contrast, in siEGR1-U251SCL (1 × 10^6^) group, only 3 mice developed xenograft tumors at 40th day. **b** Mice were euthanized and implanting tumors were harvested, and examined. siEGR1-U251-derived tumors showed smaller tumor volume compared with the volume of NC-U251-derived tumors, *P* < 0.05 *. **c** siEGR1-U251SLC-derived tumors also showed smaller tumor volume compared with the volume of NC-U251SLC-derived tumors, *P* < 0.01 **. **d** H&E staining and GFAP immunohistochemistry of siEGR1-U251-derived tumors and NC-U251-derived tumors. **e** E. Ki-67, EGR1, CCND1 staining of subcutaneous tumors in NC-U251 and siEGR1-U251 groups. Scale bar = 50 μm

Xenograft mouse model of U251 cells and U251SLCs was used to investigate the role of EGR1 on tumor growth in vivo. 5 × 10^6^ siEGR1-U251 cells and NC-U251 cells, as well as 1 × 10^6^ siEGR1-U251SLCs and NC-U251SLCs were inoculated subcutaneously into BALB/C nude mice. The mice of siEGR1-U251 and NC-U251 group developed tumors at the 50th day (Fig. 4a). In NC-U251SCL group, all mice developed xenograft tumors at Day 40. In contrast, only 3 mice developed xenograft tumors at Day 40 in siEGR1-U251SCL group (Fig. 4a). In addition, the average volumes of siEGR1-U251 tumors were approximately 1/10 of the average volumes of control (Fig. 4b). Volumes of siEGR1-U251SLC tumors also were almost 1/10 of those of control (Fig. 4c). H&E staining and GFAP Immunohistochemistry experiments revealed the xenograft tumor in mice origin of implanted U251 and U251SLC cells (Fig. 4d). Ki-67 staining showed that tumors of siEGR1-U251 group had fewer proliferative cells than NC-U251 group (Fig. 4e). EGR1 and CCND1 staining confirmed the EGR1 and CCND1 downregulation in siEGR1-U251 group (Fig. 4e).

### Overexpression of EGR1 induced by EGF enhances proliferation of glioma cells

**Fig. 5:**
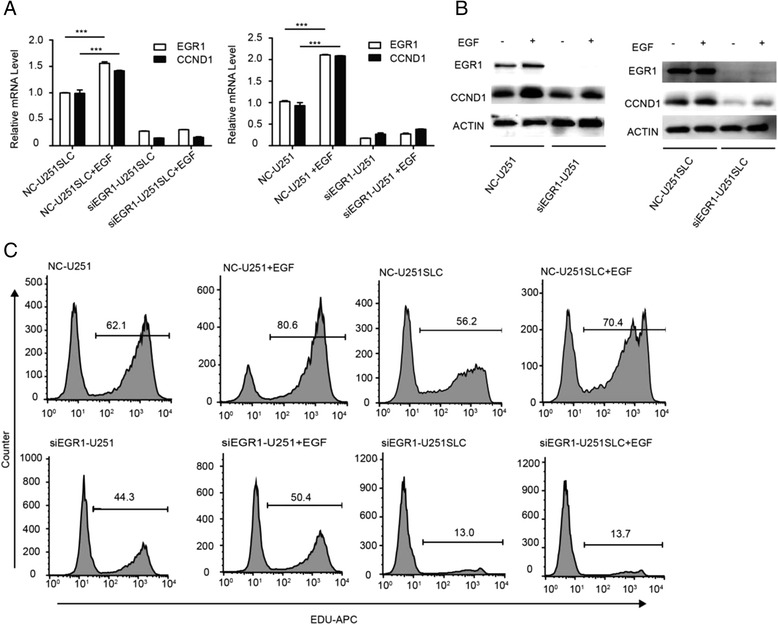
Overexpression of EGR1 induced by EGF improve proliferation of glioma cells. **a** Real-time quantitative PCR for EGR1 and CCND1 mRNA expression in U251 cells and U251SLCs after adding EGF. β-ACTIN was used as the loading control. **b** Immunoblots for EGR1 and CCND1 mRNA expression in U251 cells and U251SLCs after adding EGF. β-ACTIN was used as the loading control. **c** EdU assay for the proliferation of U251 cells group and U251SLCs group with/without EGF. *P < 0.05, **P < 0.01, ***P < 0.001 (mean ± SEM)

As shown above, knockdown of EGR1 by RNAi was able to inhibit the growth of glioma cells. We next wondered whether EGR1 over-expression promoted the growth of glioma cells. The EGF (Epidermal Growth Factor) and platelet-derived growth factor (PDGF) pathways play important roles in both CNS development and gliomagenesis, and targeted therapy against these potentially critical signaling pathways is currently under vigorous basic and clinical investigation. In glioma, EGF mainly promotes glioma cells proliferation through EGFR-MEK-ERK-ELK pathway [20]. And phosphorylated ELK1 can promote the expression of EGR1. So, in our assay, EGF (Epidermal Growth Factor) was used to induce the overexpression of EGR1 in U251 cells and U251SLCs with or without EGR1 RNAi. We found that the levels of EGR1 mRNA in U251 cells and U251SLCs reached peak (Additional file 1: Figure S3A) at 1 h after EGF treatment and began to decrease 3 h later, while the levels maintained 1.5 fold higher than the basal level until 24 h later. Consistently, the expression of CCND1 mRNA in U251 cells and U251SLCs increased to 1.5 fold at 3 h after EGF inducing, and held high levels till 24 h after EGF administration (Additional file 1: Figure S3B). Thus we chose the 6 h point for further experiments. Both EGR1 and CCND1 mRNA expression were upregulated by about 1.5 fold in U251 cells and U251SLCs by EGF (Fig. 5a). The western-blot showed that proteins of EGR1 and CCND1 increased significantly in U251 cells and U251SLCs by EGF with or without EGR1 knock-down (Fig. 5b).

EdU assay showed that the proliferation of U251 cells and U251SLCs with/without EGR1 RNAi increased along with expression up-regulation of EGR1 and CCND1 induced by EGF. The percentage of proliferation in U251 increased from 62.1% to 80.6% after EGF treatment, while the rate of proliferation increased 14.2%, 6.1% and 0.7% in U251SLCs, siEGR1-U251 cells and siEGR1-U251SLCs after EGF treatment (Fig. 5c). These findings identified that overexpression of EGR1 promoted the proliferation of glioma cells through regulating expression of CCND1.

## Discussion

EGR1, a transcription factor, controls a variety of important cellular events, such as synaptic plasticity [21], wound repair, inflammation, growth control, differentiation, apoptosis and tumor progression [22]. However, two opposing actions of EGR1, tumor suppressor and oncogene, have been described in different cancer cells. EGR1 expression is elevated in prostate cancer and prostatic adenocarcinoma cell line and contributes to proliferation, cell survival and tumor progression [23, 24]. High EGR1 expression correlates with resistance to anti-EGFR treatment in vitro and poor outcome in metastatic colorectal cancer patients treated with cetuximab [25]. On the other hand, the expression of EGR1 is frequently low in lung cancers [26], breast cancers and ovarian cancers [11, 24], which resulted in tumor suppression.

Nonetheless, EGR1 was down-regulated in glioma cells compared with normal brain tissue, its role on proliferation in glioma remains controversial. Calogero et al. reported that EGR-1 was down-regulated in dependent of ARF/Mdm2 but not p53 in human gliomas, behaving as a suppressor gene [12]. Mittelbronn et al. found that EGR1 expression was associated with enhanced patient survival in high grade astrocytomas [13]. Their results indicated that EGR1 acted as a tumor suppressor in glioma. In contrast, EGR1-expressing cells were more frequent in high grade gliomas where the nuclear expression of EGR1 was restricted to proliferating/progenitor cells. Moreover, EGR1 correlated with stemness markers and proliferation by orchestrating a PDGFA-dependent growth-stimulatory loop in primary glioma stem-like cells [14]. In the present study, we establish a stem-like cell line (U251SCL) from U251 cell lines and found that EGR1 expression was higher in U251SCL than in normal U251 cells. One report showed that there was a connection between CD133 and EGR1 and emphasized the importance of the EGR1/TCF4/CD133/LGR5 network in colorectal cancer [27]. Glioma stem-cell-like cells are mainly distinguished by CD133 and include key properties ability to a) self-renew, b) differentiate into heterogeneous types of tumor cells, and c) sustain tumor growth in vivo [28]. So, we hypothesized that growth of glioma stem-cell-like cells in vivo was relative to overexpression of EGR1. To verify our presumption, the expression of EGR1 was knockdown by RNAi in glioma cells and glioma stem-cell-like cell. The cell lines with stable suppression of EGR1 were set up. We found that the proliferation of U251SLC was inhibited remarkably by EGR1 knockdown. This result was in agreement with previous report [14]. However, Choi reported that the proliferation of U87 cells was not affected by EGR1 knockdown [29]. Did the EGR1 perform different effects on proliferation in glioma stem cells and glioma cell lines? The EGR1 knockdown of U251 and U87 cells were performed in our study. To our surprise, the proliferation of normal U251 cells and U87 cells was also reduced after knockdown EGR1 expression. It was consistent with its effect on proliferation of glioma stem-like cells. Furthermore, to observe the effect of EGR1 on growth of glioma, heterotopic mouse tumors were established from glioma U251 cells and U251SLC cells. The tumor growth was significantly inhibited in EGR1 siRNA group in both U251 and U251SLC cells. This result is in line with the phenomenon of heterotrophic mouse tumors using EGR1 knockdown mouse glioma GL261 cells [30]. Our results of xenografts further verified the inhibition of proliferation by stable knockdown EGR1 in glioma cells. And it was able to partly explain the reason why the patients with lower EGR1 expression showed longer survival. Moreover, we found that EGR1 knockdown inhibited glioma proliferation on account of G1 phase arrest, which was consistent with the study reported by Han et al. [31].

In order to further prove the promoting effect of EGR1 on proliferation, the growth factor EGF was used in our study. Through its binding to cell surface receptors, EGF is able to activate an extensive network of signal transduction pathways which include the PI3K/AKT, RAS/ERK and JAK/STAT pathways. Almost all of above pathways were able to induce the biosynthesis of EGR1 gene. For example, EGF can trigger the biosynthesis of the transcription factor EGR1 and induce proliferation via the activation of the ERK signaling pathway in astrocytes [32]. In cancer cells, the pathways which regulated cell differentiation and growth are always involved in cancer development. In lung cancer, a tight cooperation between the EGF/EGFR and mPGES-1 causes an enhanced tumorigenisis [33]. In glioma, EGF or substance P can activate EGFR, which activates ERK and EGR1 biosynthesis [34]. GBM is characterized for having a hyperactive signaling of EGFR, despite of the low expression of EGR1, its expression could be upregulated by EGFR signaling [20, 35]. In our study, EGR1 mRNA reached peak at 1 h after EGF administration and began to decrease 3 h later. This was consistent with previous reports, which showed that EGF signaling increased the EGR1 mRNA concentration in human glioma cells within 30 min. The increase of EGR1 mRNA was followed with a transient synthesis of the EGR1 protein [36]. We also found that high expression EGF can promote the proliferation of glioma cells. In HaCaT cells, EGF and thrombin triggered a rapid activation of the EGF receptor, followed by the phosphorylation and activation of ERK, which subsequently induced a transient synthesis of the EGR1, and promoted cell proliferation [37]. Although EGF can improve EGR1 expression and promote glioma cells proliferation, many articles proved that high EGR1 expression would inhibit cells growth. In our opinion, due to the stimulus diversity, similar gene expression change shows multiple effects. On the one hand, some substances that are harmful to cells can promote EGR1 expression, and inhibit cell proliferation or promote apoptosis. For example, curcumin, a natural compound, can transitorily induce expression of EGR1 and inhibits cancer cell proliferation [29]. Chlorpromazine, an antipsychotic medication, can induce expression of EGR1, thereby cause G2/M phase arrest [38]. Periplocin, a natural compound, inhibited cell viability via the ERK1/2-EGR1 pathway in vitro and in vivo [39]. On the other hand, some stimuli which are beneficial to cells also can promote the expression of EGR1 gene and cell proliferation. Growth factors and serum induce the expression of EGR1 and SRF, respectively, which in turn induces UCP expression that positively regulated cancer cell growth in HeLa cells [40]. EGF or PDGF can induce the synthesis of EGR1 via ERK signal pathway in human glioma cells, suggesting that EGR1 functions as a “third messenger” in glioma cells [36]. bFGF promotes GDNF expression accompanied with the activation of ERK5, ERK1/2 and their downstream transcription factors (c-fos, EGR1) in C6 glioma cells and results in C6 glioma cells proliferation [41]. Hence, inducible EGR1 expression in response to stress or ectopic overexpression may switch its behavior even toward an opposing effect, i.e. proliferation promotion or inhibition.

Cyclin D1(CCND1), one of three unlinked proteins (cyclin D1, D2, and D3), mainly regulates the transition of G1 to S phase during the mammalian cell cycle. The cdk/cyclin D complex regulates the phosphorylation of the retinoblastoma protein (RB) which in turn regulates proteins of the E2F family controlling the entrance of cell cycle [42]. In our assays, the proliferation inhibited by EGR1 interference was associated with the G1 phase arrest. Our results confirmed that transcription of CCND1 was directly regulated by EGR1.

## Conclusions

In conclusion, our study clarified that stable knockdown EGR1 would inhibit glioma cell growth in vitro and in vivo. The results confirmed that the basal level of high EGR1 expression will promote glioma proliferation and partly explained the reason why the patients with higher EGR1 expression showed shorter survival. The novel EGR1-CCND1 axis contributes to the G1 phase arrest and cell proliferation. The results suggest that some genes showing lower expression in cancer tissues compare with normal tissues maybe still play an important role in tumor proliferation. And further knockdown of the expression of these genes may better control the progression of cancer.

## Additional file

Additional file 1: Figure S1. Identification and characterization of U251 stem-like cells(USLC). **Figure S2.** Schematic diagram of the EGR1 binding site in CCND1 promotor. **Figure S3.** The mRNA expression of EGR1 and CCND1 at different points in time after adding EGF in U251 cells. **Figure S4.** The another two databases apart from JASPAR to corroborate the EGR1 binding site. **Figure S5.** Negative controls for the immunohistochemistry. **Figure S6** GAPDH promoter is regulated by EGR1. **Figure S7.** The positive expression control for EGR1, to validate the antibody. (DOC 2277 kb)
