# Supplementary material for: Inhibition of EGR1 inhibits glioma proliferation by targeting CCND1 promoter
Source: J Exp Clin Cancer Res. 2017 Dec 15;36:186. doi: 10.1186/s13046-017-0656-4 (PMC5732438; doi:10.1186/s13046-017-0656-4)
Supplement: Additional file 1: Figure S1. — Identification and characterization of U251 stem-like cells(USLC). Figure S2. Schematic diagram of the EGR1 binding site in CCND1 promotor. Figure S3. The mRNA expression of EGR1 and CCND1 at different points in time after adding EGF in U251 cells. Figure S4. The another two databases apart from JASPAR to corroborate the EGR1 binding site. Figure S5. Negative controls for the immunohistochemistry. Figure S6 GAPDH promoter is regulated by EGR1. Figure S7. The positive expression control for EGR1, to validate the antibody. (DOC 2277 kb) [file 13046_2017_656_MOESM1_ESM.doc]

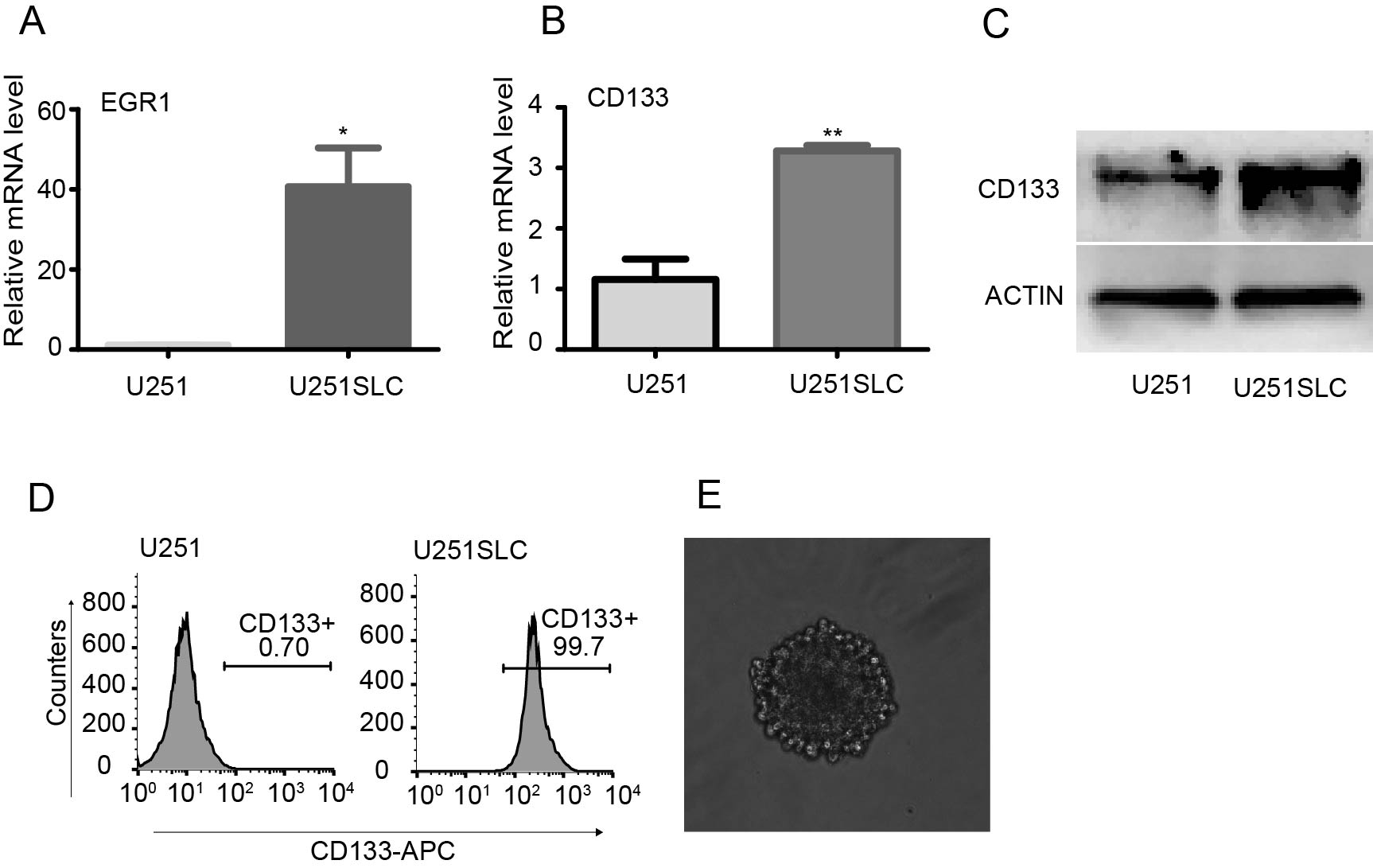
Additional file 1: Figure S1. Identification and characterization of U251 stem-like cells(USLC). (A). Real-time quantitative PCR showed higher expression of EGR1 in USLC than U251 cells. (B). Real-time quantitative PCR for CD133 in the USLC than U251 cells. β-ACTIN was used as the loading control. (C) Immunoblots for CD133 in the USLC than U251 cells. β-ACTIN was used as the loading control. (D) Flow cytometry for the proportion of CD133-positive cells in the USLC and U251 cells. (E). Subsphere derived from a single cell (magnification ×200).


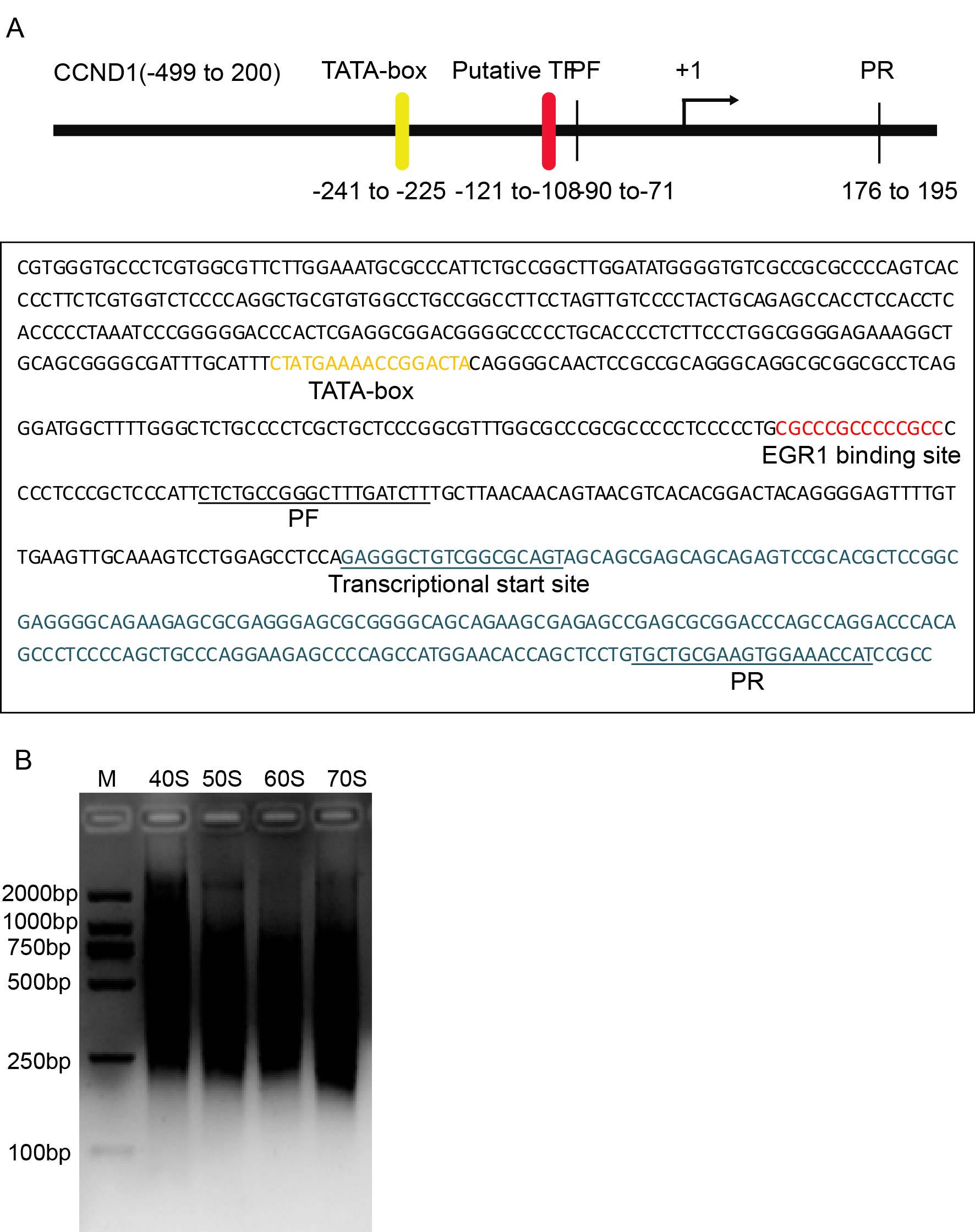


Additional file 1: Figure S2. Schematic diagram of the EGR1 binding site in CCND1 promotor. (A) CCND1 promotor sequence(-499bp to 200bp) was showed. The yellow column showed CCND1 TATA-box(-241bp to -225bp). The red column showed the EGR1 binding site(-121 to-108). The size of the primers(PF, forward primer; PR, Reverse primer) amplification was about 280bp. (B). Exploration of sonicate time. 60s was chose in present study.


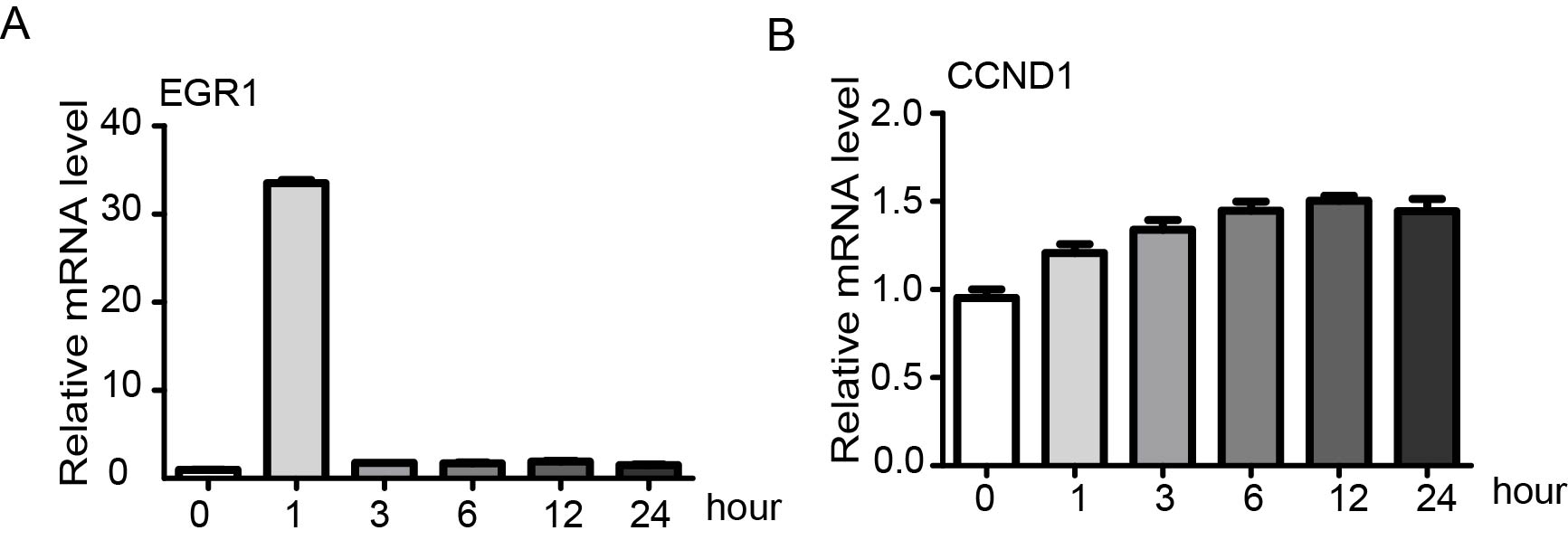


Additional file 1: Figure S3. The mRNA expression of EGR1 and CCND1 at different points in time after adding EGF in U251 cells. (A) Real-time quantitative PCR for EGR1 in U251 cells. β-ACTIN was used as the loading control. (B) Real-time quantitative PCR for CCND1 in U251 cells. β-ACTIN was used as the loading control.

A


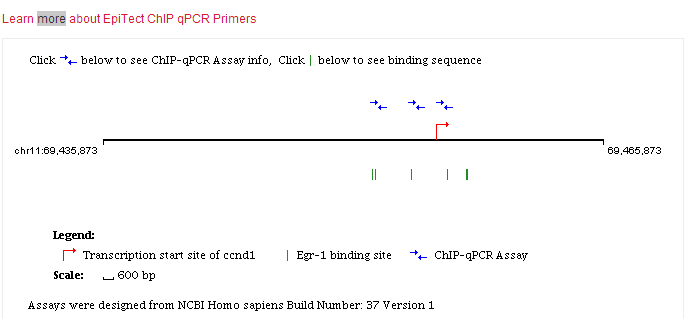


B


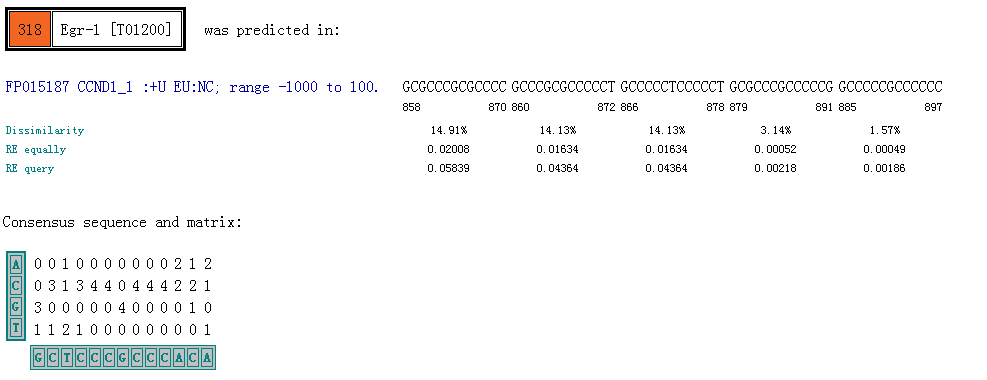


Additional file 1: Figure S4. The another two databases apart from JASPAR to corroborate the EGR1 binding site. (A). <http://www.sabiosciences.com/chipqpcrsearch.php?gene=ccnd1&species_id=0&factor=Egr-1&ninfo=n&ngene=n&nfactor=n>. (B). http://alggen.lsi.upc.es/cgi-bin/promo_v3/promo/promo.cgi?dirDB=TF_8.3&idCon=151085106800&getFile=factors/318.html

Additional file 1: Figure S5. Negative controls for the immunohistochemistry.


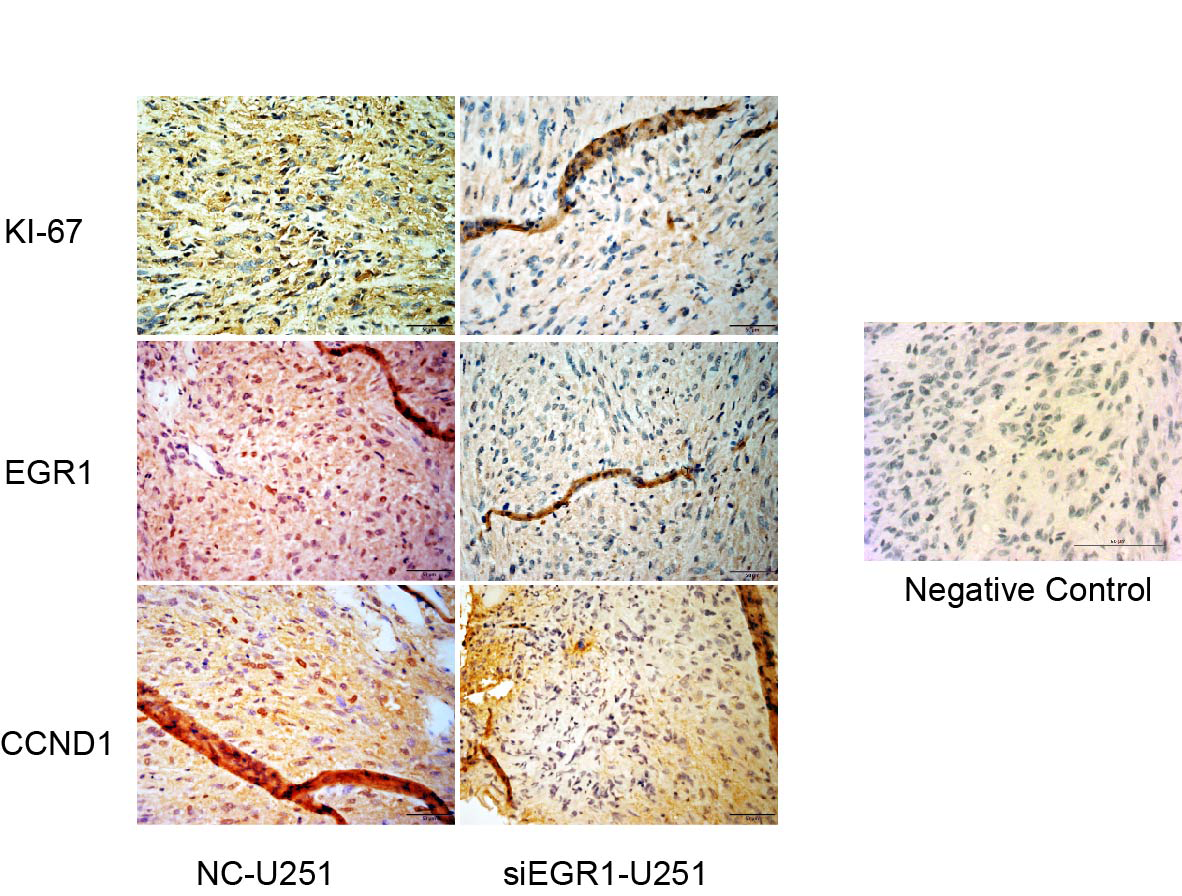

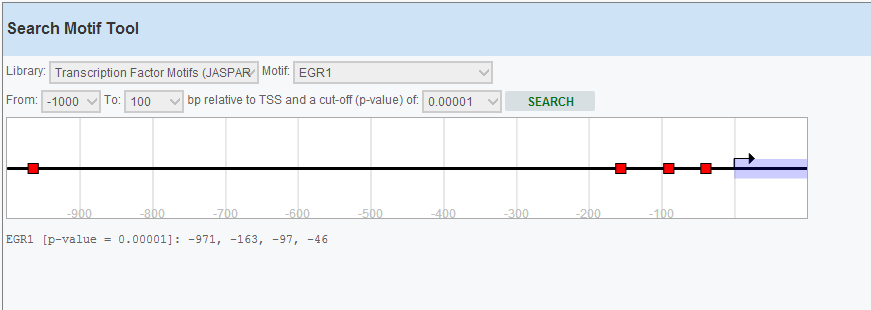


A


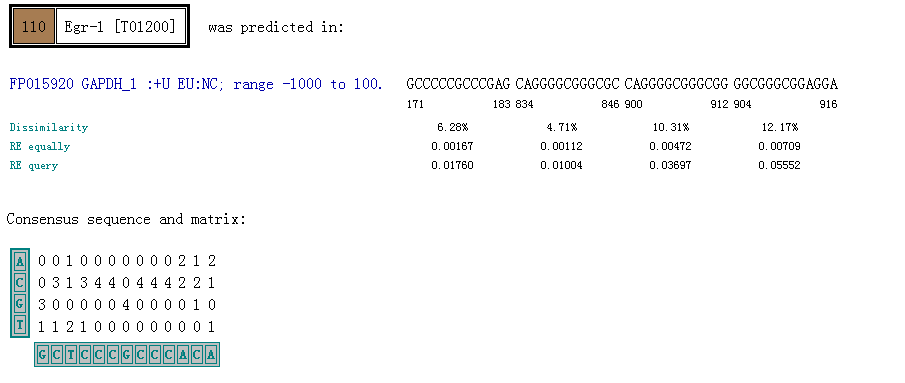


B

Additional file 1: Figure S6. GAPDH promoter is regulated by EGR1. We predicted the site in the web: (A). http://epd.vital-it.ch/cgi-bin/get_doc?db=hgEpdNew&format=genome&entry=GAPDH_1 (B). http://alggen.lsi.upc.es/cgi-bin/promo_v3/promo/promo.cgi?dirDB=TF_8.3&idCon=151063644500&getFile=factors/110.html


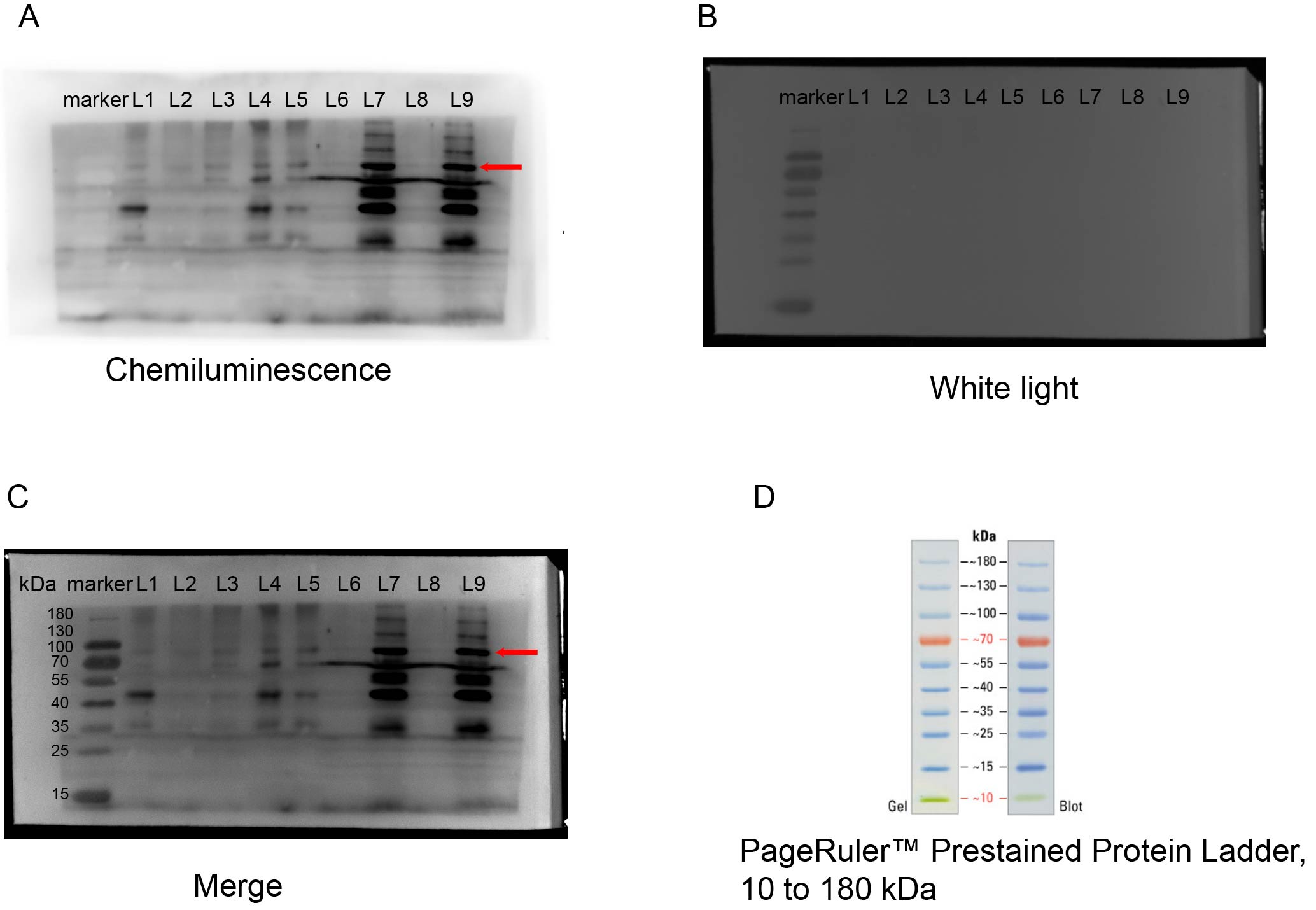


Additional file 1: Figure S7. The positive expression control for EGR1, to validate the antibody. (A), Chemiluminescence, L1-L5 are normal brain tissues. L7,L9 are positive expression control from HeLa cells lysate. The red arrow is the EGR1 strip (about 87KDa). (B). white light. White light is for display marker. (C) The merger of Chemiluminescence and white light. L7, L9 are positive expression control from HeLa cells lysate. The red arrow is the EGR1 strip (about 87 KDa). (D). Protein marker (Thermo Fisher, 26616).
